# New Insights into the Role of T3 Loop in Determining Catalytic Efficiency of GH28 Endo-Polygalacturonases

**DOI:** 10.1371/journal.pone.0135413

**Published:** 2015-09-01

**Authors:** Tao Tu, Kun Meng, Huiying Luo, Ossi Turunen, Lujia Zhang, Yanli Cheng, Xiaoyun Su, Rui Ma, Pengjun Shi, Yaru Wang, Peilong Yang, Bin Yao

**Affiliations:** 1 Key Laboratory for Feed Biotechnology of the Ministry of Agriculture, Feed Research Institute, Chinese Academy of Agricultural Sciences, Beijing, 100081, P. R. China; 2 Department of Biotechnology and Chemical Technology, School of Chemical Technology, Aalto University, FI-00076, Aalto, Finland; 3 State Key Laboratory of Bioreactor Engineering, East China University of Science and Technology, Shanghai, 200237, P. R. China; University of Canterbury, NEW ZEALAND

## Abstract

Intramolecular mobility and conformational changes of flexible loops have important roles in the structural and functional integrity of proteins. The *Achaetomium* sp. Xz8 endo-polygalacturonase (PG8fn) of glycoside hydrolase (GH) family 28 is distinguished for its high catalytic activity (28,000 U/mg). Structure modeling indicated that PG8fn has a flexible T3 loop that folds partly above the substrate in the active site, and forms a hydrogen bond to the substrate by a highly conserved residue Asn94 in the active site cleft. Our research investigates the catalytic roles of Asn94 in T3 loop which is located above the catalytic residues on one side of the substrate. Molecular dynamics simulation performed on the mutant N94A revealed the loss of the hydrogen bond formed by the hydroxyl group at O34 of pentagalacturonic acid and the crucial ND2 of Asn94 and the consequent detachment and rotation of the substrate away from the active site, and that on N94Q caused the substrate to drift away from its place due to the longer side chain. In line with the simulations, site-directed mutagenesis at this site showed that this position is very sensitive to amino acid substitutions. Except for the altered *K*_*m*_ values from 0.32 (wild type PG8fn) to 0.75–4.74 mg/ml, all mutants displayed remarkably lowered *k*_*cat*_ (~3–20,000 fold) and *k*_*cat*_/*K*_*m*_ (~8–187,500 fold) values and significantly increased △(△G) values (5.92–33.47 kJ/mol). Taken together, Asn94 in the GH28 T3 loop has a critical role in positioning the substrate in a correct way close to the catalytic residues.

## Introduction

Pectin is a family of very complex polysaccharides that is mainly located in the middle lamella and primary cell walls of terrestrial plants, accounting for one-third of the plant dry weight [1]. It is composed of long galacturonic acid chains with carboxyl groups and varying methyl ester contents [2, 3]. Because of the complexity of pectin, its degradation is facilitated by a battery of pectinases, including polygalacturonase (PG), pectate lyase, rhamnogalacturonase, pectin methylesterase, and pectin acetylesterase [4]. Pectinolytic enzymes are widely used in the fruit juice, paper, and textile industries as well as the extraction of oils [5]. One of the most studied and widely used commercial pectinases is PG [6]. Whatever the targeted application, insight to the catalytic properties of PG is an overarching aim and worthy of extensive study. Therefore, the knowledge of the structure and function of PGs is of great importance to understand the catalytic mechanism.

**Fig 1:**
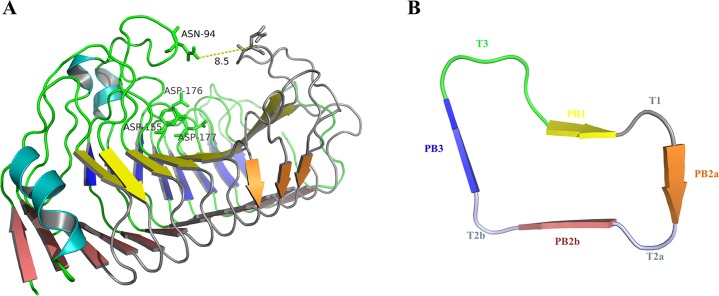
Structure analysis of wild type PG8fn. (A) Schematic structure of PG8fn with the N-terminus on the left and the C-terminus on the right viewed onto β-sheet PB1 (yellow). PB2a, PB2b, and PB3 are shown in orange, pink, and blue, respectively. The key residues, Asn94, Asp155, Asp176, and Asp177, are shown in stick models. The structure viewed from the N-terminal side shows the cleft that is formed by the loops T1 (right side) and T3 (left side). (B) Ribbon diagram of the cross section of PG8fn on the right panel; the color code indicates the secondary structure elements in a representative coil.

The endo-PGs have been receiving much attention as a major group of pectinase due to their capability of removing spoilage and decay of the processed foods [6]. Most of the endo-PGs have been classified into glycosyl hydrolase (GH) family 28 based on amino acid sequence similarity analysis [7]. So far, ten structures of GH28 members have been resolved, including PehA from *Erwinia carotovora* (PDB: 1BHE) [8], Endo-PG II (1CZF) [9] and Endo-PG I (1NHC) [10] from *Aspergillus niger*, FmPG from *Fusarium moniliforme* (1HG8) [11], PGA from *Aspergillus aculeatus* (1IA5/1IB4) [12], Endo-PG I from *Stereum purpureum* (1K5C/1KCC/1KCD) [13], and CluPG1 from *Colletotrichum lupini* (2IQ7) [14]. The catalytic domain of these enzymes share a common structure: a right-handed parallel β-helical architecture made up of ten complete coils (Fig 1A). Each coil of β-helix is formed by three or four β-strands developing four parallel β sheets (named PB1, PB2a, PB2b, and PB3), and three or four loops connecting β-strands (named T1, T2a, T2b, and T3 correspondingly) (Fig 1B). The β-strand PB1 forms the relatively flat bottom of the cleft [14], and the loops T3 and T1 in close proximity to the N- and C-terminus of the β-helical architecture, respectively, form two bulky extensions on the exterior surface of the slightly twisted β-helix and wrap the long cleft. The substrate binding pocket (SBP) is oriented obliquely to the helical axis of the β-helix, well suited to accommodate the unbranched substrate, and is open at both ends in accordance with the endo-hydrolytic character of the enzyme [12].

Due to the efficient, specific, cost-effective and environmentally friendly characteristics of enzyme-catalyzed reactions, enzymes have aroused general interest in the study of catalysis. The functions of enzymes are intimately related to protein flexibility and conformation of some structural elements [15, 16]. As the most flexible parts of protein structures [17], loop regions have been shown to involve the catalytic activity of enzymes and play major roles in substrate selectivity and recognition by facilitating substrate binding to the cleft [18–22]. However, the exact roles of the active site loops of GH28 endo-PGs are unknown yet. The crystal structure of *C*. *lupini* CluPG1 (2IQ7) [14] shows seven molecules in the asymmetric unit with well-superimposed conformations including the T3 and T1 loops (see S1 Fig). Interestingly, Lys114 and Lys124 on the T3 loop, and Glu290 and Asn291 on the T1 loop are highly mobile. What’s more, comparison of the three crystal structures (1K5C/1KCC/1KCD) of *S*. *purpureum* endo-PG I in native and two galacturonic acid complex states at atomic resolution [13] showed that the conformation of the side chain of Asn91 (corresponding to Asn117 in 2IQ7) changed slightly due to the hydrogen bonding network formed around the bound galacturonic acid (GalpA) molecule (see S2 Fig). It indicated that the key tip residue of T3 loop, Asn, might play a key role in substrate binding for catalytic reaction. Similar findings have been reported in a previous study that the Ala143 of GTPase in the G5 loop plays an important role in the nucleotide release rate by allowing the correct positioning and hydrogen bonding [15].

In our previous studies, a GH28 endo-PG, PG8fn, was identified from *Achaetomium* sp. Xz8 [23]. It is distinguished from other endo-PGs by its high specific activity (28,000 U/mg) towards polygalacturonic acid. Structural and sequence analysis showed that the T3 loop of PG8fn is located at the entry and exit of the catalytic cleft and the highly conserved residue Asn94 (corresponding to Asn91 in 1K5C and Asn117 in 2IQ7) forms a hydrogen bond to the substrate in the active site cleft. To verify the functions of T3 loop and Asn94 in it, molecular docking studies and molecular dynamics (MD) simulations were carried out to determine the role of T3 loop in the enzyme-substrate interaction, and site-directed mutagenesis was employed to substitute Asn94 with other amino acids (Asp, Leu, Cys, Gln, Gly, Ser, Ala) taking into account the steric factors. Further kinetic studies gave insights into the role of this key site (Asn94) in substrate binding and catalytic reaction.

## Materials and Methods

### Chemicals, plasmids and strains

The substrate polygalacturonic acid was purchased from Sigma-Aldrich (St. Louis, MO). All other standard chemicals were of analytical grade. *Achaetomium* sp. Xz8 CGMCC6545 (China General Microbiological Culture Collection Center, Beijing, China) was the donor of the GH28 endo-PG gene (*pg8fn*, KC633130) [23]. *Escherichia coli* Trans I-T1 and the pEASY-T3 vector (TransGen, Beijing, China) were used for plasmid amplification and construction, respectively. Plasmid pPIC9 and *Pichia pastoris* GS115 from Invitrogen (Carlsbad, CA) were used for heterologous expression. Enzymes FastPfu DNA polymerase from TransGen and restriction endonucleases and T4 DNA ligase from New England BioLabs (Hitchin, UK) were also purchased.

### Binding conformation determination

The initial structure of the wild type PG8fn was obtained by automated homology-modeling from Swiss-Model server with the crystal structure of endo-PG from *C*. *lupini* (2IQ7_A, 79.6% identity) as template. To remove steric clashes, the model with the lowest value of MOLPdf was selected and refined by energy minimization using the YASARA software (www.yasara.org). The quality of the refined structure was evaluated by PROCHECK and Verify3D [24, 25]. To perform docking studies, the coordinate files of ligand were prepared in the PRODRG server (http://davapc1.bioch.dundee.ac.uk/cgi-bin/prodrg). The docking of enzyme and pentagalacturonic acid was carried out by AutoDock Vina [26]. In comparison to AutoDock 4, AutoDock Vina employs the Lamarckian genetic algorithm and simulated annealing approach to explore the full range of ligand conformational flexibility and the rotational flexibility of selected receptor hydrogens and significantly improves the average accuracy of the binding mode predictions. A docking grid with a size of 60 Å × 60 Å × 60 Å was used to encompass the ligand-partial protein complex. The center of the box was set at the C_α_ atom of Lys233, the important contributor to recognize the carboxyl group of the substrate [13]. The set number generated for the docking poses was 20. The docked complex was selected by the criteria of interacting energy combined with the geometrical matching quality and the modeled structure of the polygalacturonase-octagalacturonate complex [12], and the energy was minimized using YASARA software to resolve atomic clashes. The docking results were visualized using PyMOL (The PyMOL Molecular Graphics System, Delano Scientific, Portland, OR).

### MD simulation

To explain the effect of T3 loop on the catalytic efficiency of PG8fn, MD simulation was performed to investigate the dynamic properties of four systems: a control system without substrate of apo monomeric wild type PG8fn, a system with pentagalacturonic acid bound to the catalytic pocket of wild type PG8fn, and the system with pentagalacturonic acid bound to the catalytic pocket of mutants (N94Q or N94A). In order to have the same starting substrate structure for the wild type and mutants, the wild type protein complex was submitted to *in silico* mutagenesis (N94Q or N94A) with Discovery Studio 4.1 (Accelrys Software, San Diego, CA). MD simulation was performed with the GROMACS 4.5.5 package and protein atoms with AMBER99SB force field [27]. The program Antechamber was used to generate the GLYCAM06 force field parameters [28] with the AMBER 11 simulation package following the protocol of Mercadante et al. [29]. Each simulation was repeated three times with the same initial configurations. Structures of the enzyme or enzyme-substrate complexes were immersed in a rectangular cubic box of transferable intermolecular potential 3p (TIP3p) water as the solvent. To obtain a neutral charge of the simulated systems, eleven chloride-ions were included. Each system was subjected to energy minimization for 10,000 steps by steepest descents. Long range electrostatic interactions were predicated using the particle mesh Ewald (PME) method. Following the minimization, position restrained MD simulation was carried out upon slow heating to 300 K in an NPT ensemble with a constant number of particles (N), constant system pressure (P), and constant temperature (T) at 1 atm pressure over a period of 500 ps. The final production phase of simulations was then carried out for a total of 50 ns MD simulation with a time step of 1 fs at the constant pressure (1 atm) and temperature (300 K). Analyses of MD trajectories were carried out employing VMD [30] and its plugins. The root mean square deviation (RMSD) was calculated for the protein backbone atoms using least-squares fitting, and the root mean square fluctuation (RMSF) was calculated using the coordinates derived from the MD trajectories of the last 20 ns timescale. Putative hydrogen bonds were assigned based on two geometric criteria for each trajectory frame saved: the distance of less than 2.5 Å and the angle larger than 120 degrees between the acceptor and hydrogen donor.

### T3 loop sequence analysis

GH28 endo-PGs sharing more than 30% identities with PG8fn were collected from a blast analysis (http://www.uniprot.org/blast), and aligned using the ClustalX 2.1. Using 2IQ7_A as the template (79.4% identity), the tertiary structure of PG8fn was predicted, and the approximate position of T3 loop was determined. T3 loops of other GH28 family members were identified with PG8fn as reference using the sequence display function on the PDB database website (http://www.rcsb.org/pdb/).

### Construction of PG8fn mutants

Based on the structural and sequence analysis, the key residue related to the role of T3 loop, Asn94, was determined. The wild type PG gene *pg8fn* was used as the DNA template. Site-directed mutagenesis was performed to replace Asn94 by overlap extension PCR [31] with primers listed in S1 Table. The mutant gene fragments were inserted into pPIC9 vector at *Sna*BI and *Not*I sites. Plasmids harboring desired mutations were identified by DNA sequencing, and the recombinant plasmids containing the correct mutant genes were linearized with *Bgl*II and then individually transformed into *P*. *pastoris* GS115 competent cells by electroporation. The positive transformants with methanol-utilizing ability were identified on minimal methanol or minimal dextrose medium agar plates according to the supplier’s protocol (Invitrogen).

### Purification of wild type PG8fn mutants

The positive transformants with highest endo-PG activity were selected for fermentation in 1-l conical flasks following the *Pichia* Expression Kit instructions (Invitrogen). The supernatants (~1 l) of the induced cultures were collected by centrifugation at 12,000 ×*g*, 4°C for 10 min to remove cell debris and undissolved materials. The cell-free supernatants were concentrated through a 10 kDa cutoff Vivaflow 200 ultrafiltration membrane (Vivascience, Hannova, Germany), and then loaded on HiTrap desalting column (GE Healthcare, Uppsala, Sweden), Amicon Ultra Centrifugal Filter Device PL-10 (Millipore, Billerica, MA, USA) and HiTrap SP XL 5 ml FPLC column (GE Healthcare) on an ÄKTA Avant 25 system for further purification.

The molecular weights and purities of the purified proteins were analyzed by sodium dodecyl sulfate-polyacrylamide gel electrophoresis (SDS-PAGE) in a 12.0% gel. The protein concentrations were determined using the Bio-Rad protein assay kit (Boston, MA). *N*-deglycosylation was performed according to the method described previously [32] and then analyzed by SDS-PAGE.

### Enzyme and kinetic assays

The PG activity was determined using the 3,5-dinitrosalicylic acid (DNS) method [33] and d-(+)-galacturonic acid was used as the standard. The standard reaction mixture consisting of 900 μl of 0.33% (w/v) polygalacturonic acid in McIlvaine buffer (200 mM Na_2_HPO_4_, 100 mM citric acid, pH 6.0) and 100 μl of appropriately diluted enzyme solution was incubated at 45°C for 10 min, and then the reaction was stopped with 1.5 ml of DNS reagent followed by 5 min boiling. After cooling down to room temperature, its absorption at 540 nm was measured. One unit (U) of endo-PG activity was defined as the amount of enzyme that released reducing sugars equivalent to 1 μmol of d-(+)-galacturonic acid per min under standard conditions (pH 6.0, 45°C, 10 min). Each reaction and its controls were run in triplicate.

Kinetic parameters including *K*_*m*_ (mg/ml), maximum velocity (*V*_*max*_, μmol/min/mg), and *k*_*cat*_ (/s) were determined in McIlvaine buffer (pH 6.0) containing 0.1–10 mg/ml polygalacturonic acid at 45°C for 5 min. The kinetic values were determined by the GraFit7 Setup software (Biosoft, Cambridge, UK). The experiments were carried out three times, and each experiment included triplicates. The data represent the average of all statistically relevant data with a standard deviation of less than 10%.

## Results and Discussion

### Determination of binding conformation

**Fig 2:**
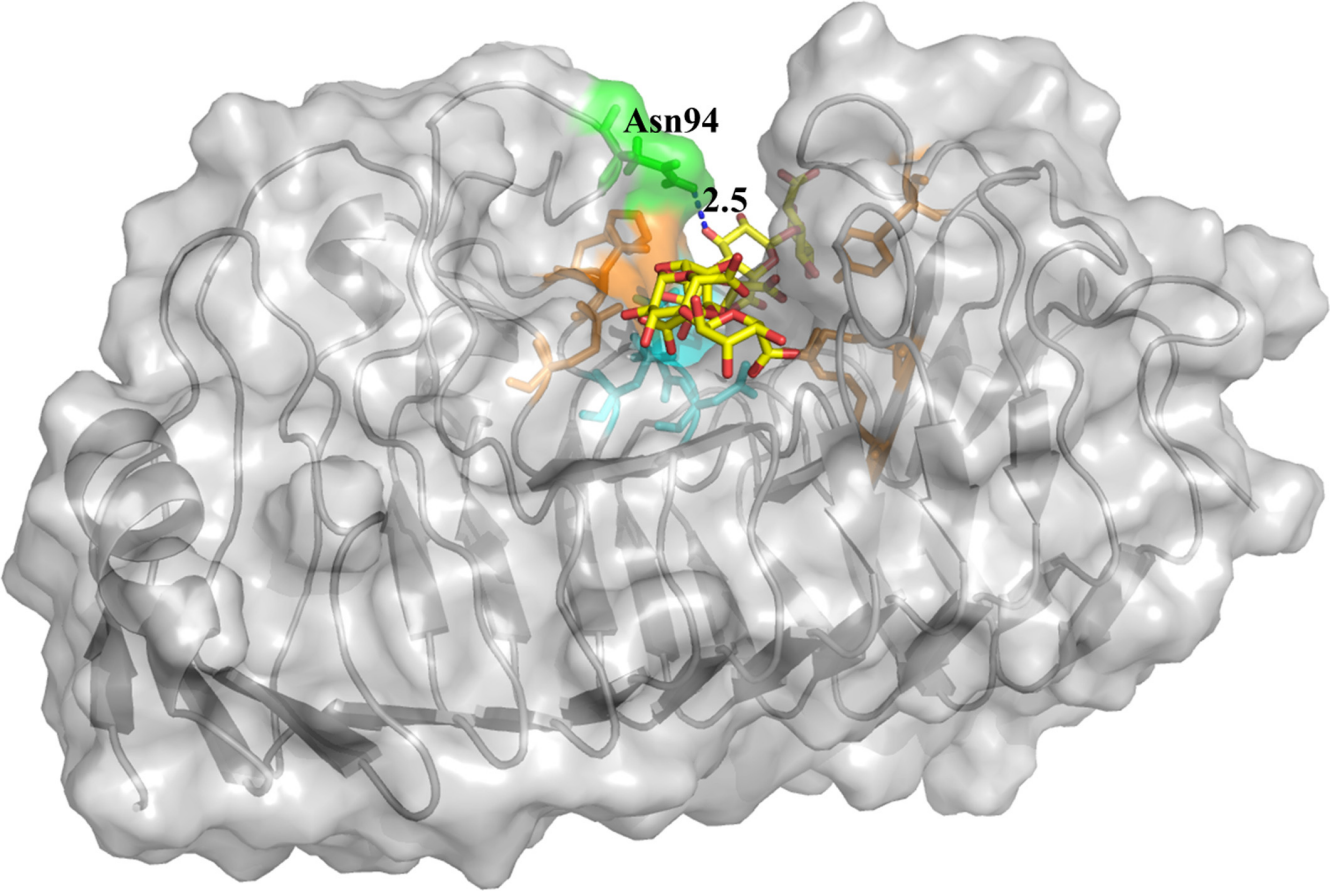
Illustration of the substrate pentagalacturonic acid docked to the wild type PG8fn catalytic pocket. The system was constructed using PyMOL. The protein surface is shown in transparent gray. The catalytic region forms a tunnel through which the substrate passes. Hydrogen bond is depicted as blue dashed lines. Asn94 is marked in green. Catalytic triads in the active center are marked in cyan. The key amino acids interacted with GalpA at –1/+1 subsites are marked in orange.

For elucidation of the functional role of the mobile T3 loop of PG8fn in the recognition of the substrate, molecular docking studies were performed. The structure of PG8fn was modified through energy minimization, and the quality validation indicated that the comparative model is sufficient for further analysis. The docking results are listed in S2 Table. The predicted binding affinity of all binding modes is in the range from −9.3 to −8.0 kcal/mol, and the substrate pentagalacturonic acid has a very flexible conformation as shown by the changing calculated RMSD values relative to the best mode. After subsequent equilibration stable positions for the substrate were established, the conformation of PG8fn-pentagalacturonic acid complex (Fig 2) was generated based on the conformation of galacturonate units co-crystallized with *S*. *purpureum* endo-PG I (1KCD) [13] and the modeled structure of the polygalacturonase-octagalacturonate complex [12]. The active site of PG8fn is located at the bottom of a shallow groove enclosed by the β-strand PB1 and T1 and T3 loops. The pentagalacturonic acid docked into the SBP of the wild type PG8fn showed an extended conformation, representing a highly stable enzyme-substrate complex to allow the formation of productive complex and formation of the glycosyl-enzyme intermediate. According to the putative structure of PG8fn, Asp155, Asp176 and Asp177 form the catalytic triads corresponding to Asp153, Asp173 and Asp174 in *S*. *purpureum* endo-PG I [13], respectively. Therefore, Asp176 is the putative acid/base and Asp177 and Asp155 activate the water molecule that functions as a nucleophile in the inverting mechanism. Asp177 and Asp155 are closely located at the bottom of the active site with a separation of less than 5.0 Å, similar to that of *A*. *aculeatus* PG [12]. The non-reducing end of the substrate is directed toward the N terminus of the enzyme according to Pagès et al. [34]. GalpA at subsite –1/+1 interacts with Asn94, Gln124, His152, Asn153, Arg231, Lys233, and Tyr266 by forming hydrogen bonds. The O34 hydroxyl group of pentagalacturonic acid at the subsite +1 forms a hydrogen bond with the ND2 of Asn94 (2.5 Å), which is consistent with the crystal structures of *S*. *purpureum* endo-PG I complex [13]. These results highlight the important role of Asn94 of T3 loop in substrate binding.

### Conformation analysis of MD simulation

To further investigate the structural role of residue 94 in T3 loop region at the entry and exit of the catalytic cleft, the wild type PG8fn complex was submitted to *in silico* mutagenesis. Two different residues (Gln and Ala) were incorporated at position 94. Mutation to Gln having similar but longer side chain as Asn could provide information on the effect of the side chain length on the correct positioning of the substrate. Mutation to Ala was used to study the effect of short side chain without hydrogen bonding ability on the positioning of the substrate and the effect on catalysis. MD simulation of the wild type and mutant enzymes with the substrate in the active site (PG8fn/N94Q/N94A-pentagalacturonic acid) were used to analyze the effect of mutations on ligand binding and plasticity of protein.

**Fig 3:**
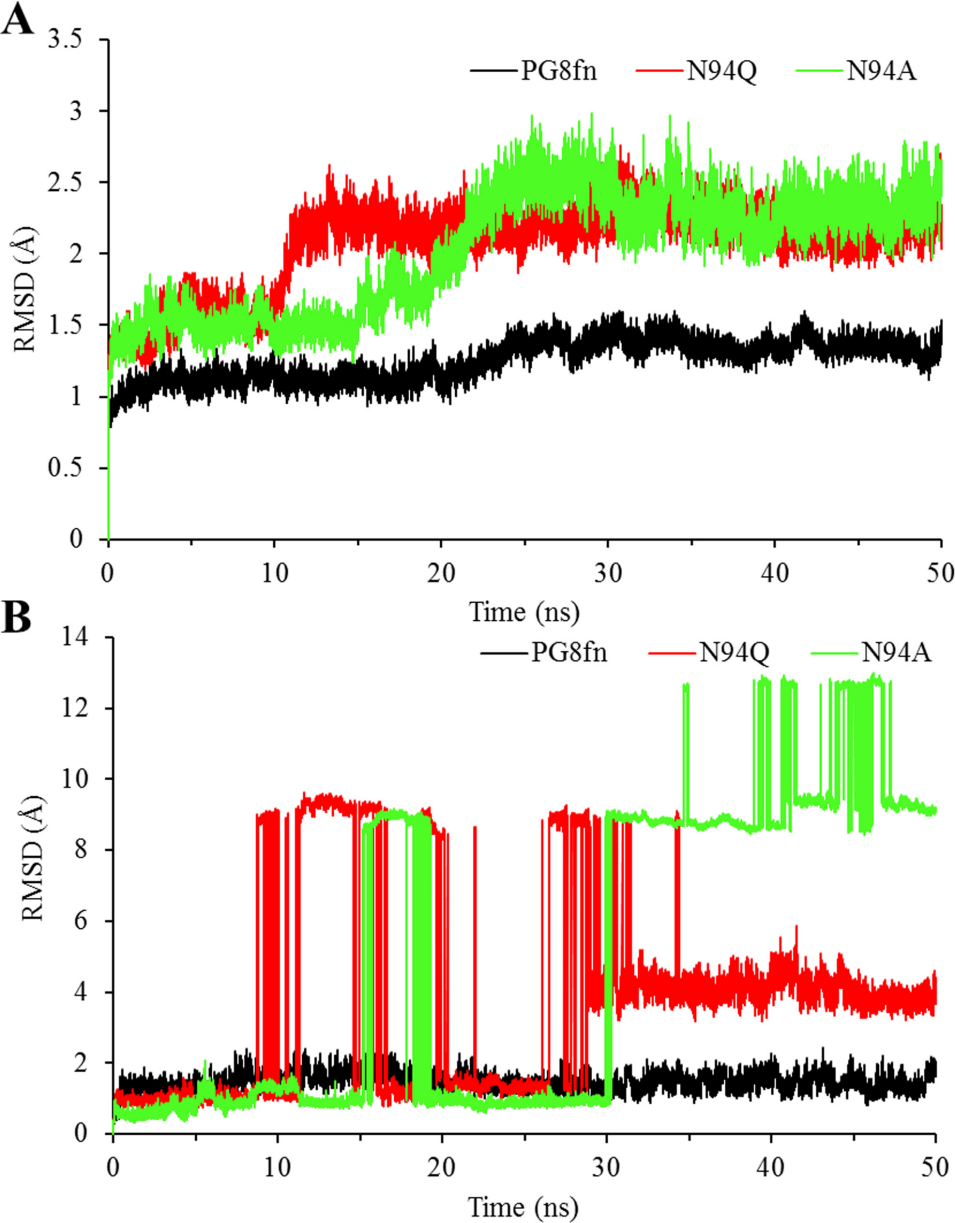
Root mean square deviation (RMSD) analysis of the wild type PG8fn (black), N94Q (red) and N94A (green) during a 50-ns MD simulation. (A) The enzyme backbones. (B) The pentagalacturonic acid. Each simulation was repeated three times with the same initial configurations.

The RMSD values of the protein backbone atoms against the starting structures during the full MD simulation showed that all complex systems became dynamically equilibrated after 30 ns of simulation (Fig 3A). Both substitutions showed increased conformational flexibility with higher RMSD values at the temperature of 300 K. The two abrupt changes in RMSD values of the two mutant complexes are related to the equilibration protocol, in which the entire protein backbone had its position constrained initially, but the atoms of the protein backbone close to the substrate remained constrained in the second step only. The ligand RMSD depicted in Fig 3B showed that the pentagalacturonic acid in both mutant complexes is much more flexible in the catalytic site than that in the wild type. It’s the substrate detachment and rotation to account for the dissociation of pentagalacturonic acid from the protein.

**Fig 4:**
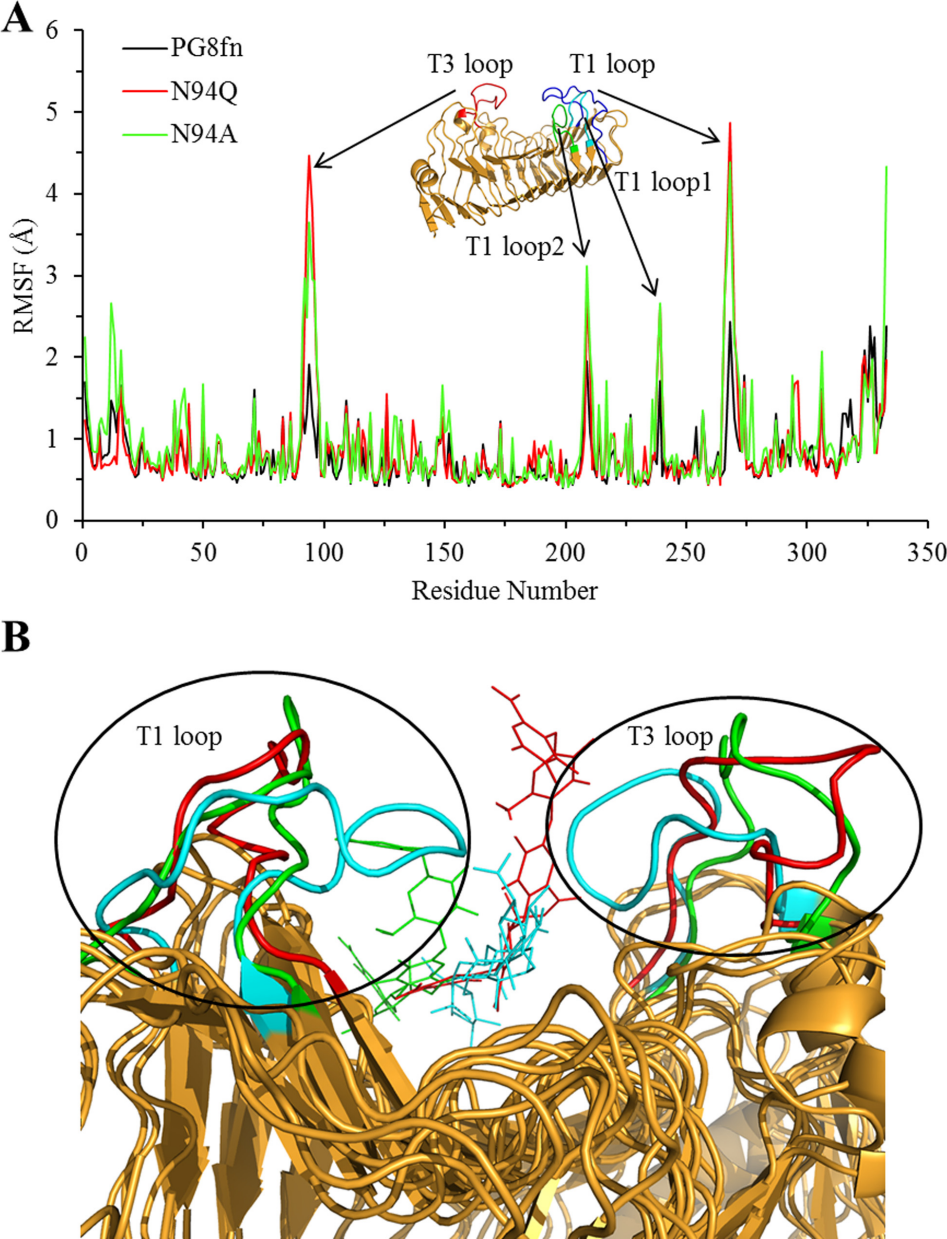
Conformation analysis of the last 20 ns MD trajectory of the enzymes. (A) The RMSF value of each residue C_α_ atom of the wild type PG8fn (black) and mutants N94Q (red) and N94A (green) measured against the corresponding starting structures. (B) Superimpositions of the average structure of the wild type PG8fn and two mutants viewed from the C-terminal side.

The stabilized RMSD values of the last 20 ns indicated the reliability and suitability of these MD trajectories for further analysis. The RMSF value of each residue C_α_ atom was also calculated with respect to the starting structures (Fig 4A). Significant differences were detected in the fluctuations of T3 and T1 loops of wild type PG8fn and its two mutants: that in the mutant systems are more flexible than in the wild type. Distinct conformations of T3 and T1 loops were obtained by clustering the last 20 ns MD trajectories and superimposing the average structure of the wild type PG8fn and the two mutants (Fig 4B). For all systems, the conformations superimposed well except in the flexible regions, especially the T3 and T1 loops. In the wild type PG8fn, the ND2 of Asn94 formed a hydrogen bond with the O34 hydroxyl group of pentagalacturonic acid in the +1 subsite during the simulation. This hydrogen bond interaction not only plays an important role in stabilizing the substrate, but also contributes to keep the substrate in a position accessible to catalytic residues. In contrast, no similar hydrogen bonds formed in the mutants N94Q and N94A. As results, the flexibility of T3 and T1 loops of mutants enhanced and the pentagalacturonic acid was dissociated from the SBP, i.e. substrate detachment and rotation. The results revealed the important role of Asn94 on T3 loop for substrate binding and locating to ensure efficient catalytic reactions.

In order to investigate the flexibility changes of T3 loop after mutation, putative hydrogen bonds were predicted for the last 20 ns of MD simulations. Overall, 16 hydrogen bonds existed in the T3 loop (residues 88−101) of the wild type PG8fn, and 14 and 10 hydrogen bonds for mutants N94Q and N94A, respectively (Table 1). The hydrogen bond occupancy involving N94 was above 90%, and the hydrogen bond formed by S93 in PG8fn was not observed in the two mutants. Such weaker hydrogen bond interactions may contribute to the increased flexibility of T3 loop in the two mutants. What’s more, K91 on T3 loop of N94Q formed hydrogen bonds with A84 and Q149, facilitating the movement of T3 loop away from T1 loop, resulting in the dissociation of the substrate from the enzyme.

**Table 1:** Hydrogen bonds^a^ existing in T3 loop (residues from 88 to 101) of wild type PG8fn and two mutants (N94Q, N94A), and their occupancies during the last 20 ns of MD simulations.

Wild type			N94Q			N94A		
Donor	Acceptor	Occupancy (%)	Donor	Acceptor	Occupancy (%)	Donor	Acceptor	Occupancy (%)
G78@O	W88@N	99.89	G78@O	W88@N	97.35	G84@O	W88@N	98.54
T121@O	W88@NE1	86.47	T121@O	W88@NE1	88.91	A85@O	W88@N	90.01
P122@O	W88@NE1	98.76	P122@O	W88@NE1	95.45	P122@O	W88@NE1	92.33
—^*b*^	—	—	D89@OD2	N153@ND2	57.82	—	—	—
G150@O	T90@N	87.54	N153@OD1	T90@N	68.74	—	—	—
—	—	—	A85@O	K91@NZ	71.34	—	—	—
—	—	—	Q149@O	K91@NZ	69.58	—	—	—
—	—	—	G150@O	K91@NZ	75.24	—	—	—
S93@OG	Q124@NE2	89.84	—	—	—	—	—	—
N153@OD1	S93@N	83.65	—	—	—	—	—	—
GalpA^+1^@O34	N94@ND2	90.31	—	—	—	—	—	—
K86@O	K97@NZ	88.76	K86@O	K97@NZ	89.37	K86@O	K97@NZ	79.93
Y55@OH	K97@NZ	57.68	Y55@OH	K97@NZ	63.56	Y55@OH	K97@NZ	68.75
E57@OE1	K98@N	55.79	E57@OE1	K98@N	61.28	K98@O	W87@NE1	50.08
K98@O	W58@N	52.87	K98@O	W58@N	59.60	K98@O	W58@N	68.17
—	—	—	—	—	—	N153@OD1	K99@NZ	62.58
GalpA^-2^@O17	K101@NZ	78.64	—	—	—	—	—	—
GalpA^-3^@O6	K101@NZ	65.73	GalpA^-3^@O6	K101@NZ	80.33	—	—	—
D158@OD1	K101@NZ	69.36	D158@OD1	K101@NZ	73.05	—	—	—
A59@O	K101@N	80.46	—	—	—	A59@O	K101@N	76.36
K101@O	L62@N	74.58	—	—	—	K101@O	L62@N	68.09

^*a*^ Only H-bonds with occupancies >50% are shown.

^*b*^ Not observed.

### Analysis of sequence conservation in T3 loop

**Fig 5:**
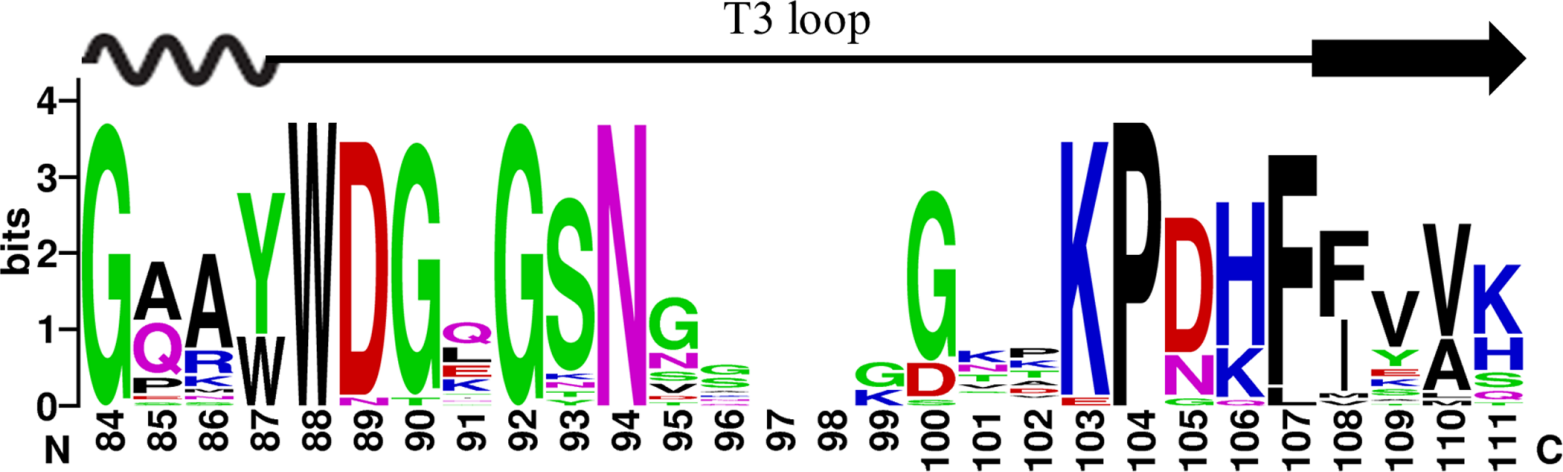
The consensus sequence logo of the loop T3 regions of 1,000 GH28 endo-PGs generated using the WEBLOGO.

According to the statistics drawn from the carbohydrate-active enzyme (CAZy) database [35], GH28 consists of 2,547 members from both microorganisms (archaea, bacteria and microbial eukaryotes) and plants. The vast majority of GH28 enzymes appear to be PGs, and 205 of them have been biochemically characterized. Using the protein sequence of PG8fn as a query, the Blast search generated a group of 1,000 complete sequences encoding putative GH28 endo-PGs of fungi. Further analysis of this GH28 subset revealed that the amino acid residue occupying position 94 is highly conserved as Asn in the T3 loop region (Fig 5), which is among the most flexible regions in PG [36]. The results indicated that the conserved residue of T3 loop, Asn94, might play an important role in the catalytic process. It is widely acknowledged that mobile loops in close proximity to the active site demonstrate pronounced conformational flexibility and contribute to enzyme catalysis. For example, the conformational change of a highly conserved thumb-loop of the GH11 xylanase close to the active site is directly associated with the binding of the substrate and the release of the product [19–21, 37–41]. In other words, the importance of the amino acid side chain at position 94 might be comparable to that of Ile116 at the tip of the thumb-loop of GH11 xylanase from *Thermobacillus xylanilyticus* [19–21], and that of W99 in the β2α2 loop region of GH51 arabinofuranosidase from *T*. *xylanilyticus* [22]. This is the first report on the role of T3 loop in the catalytic efficiency of GH28 endo-PG. Therefore, considering the above analysis, the residue Asn94 is an important position to investigate the effect of mutations on the catalytic efficiency.

### Expression and purification of wild type PG8fn and its mutants

We analyzed how the biochemical properties of various amino acids at position 94 affect the catalytic properties by replacing it with Ala, Cys, Gly, Ser, Leu, Gln and Asp, respectively. These side-chain alterations were chosen, not only because they represent variability in biochemical properties and spatial occupancy, but also because none of these side-chains naturally occurs at position 94 in known GH28 endo-PGs.

Wild type PG8fn and its mutants were generated and expressed in *P*. *pastoris* and purified to electrophoretic homogeneity by one-step cation exchange chromatography. SDS-PAGE revealed that all recombinant enzymes had apparent molecular masses of ~38 to 42 kDa, which were higher than their calculated molecular masses (35.4 kDa). After treatment with Endo H, all enzymes showed a single band corresponding to the theoretical mass (see S3 Fig).

### Kinetic analysis of wild type PG8fn and its mutants

The kinetic parameters were determined for the wild type PG8fn and its mutants with polygalacturonic acid as the substrate (Table 2). Compared with the high specific activity of the wild type PG8fn (28,000 U/mg), all mutants displayed reduced specific activity, especially N94S and N94A, which lost almost completely their activity. Furthermore, all mutants showed significantly altered kinetic parameters. The *K*_*m*_ values of all mutants increased from 0.32 mg/ml of the wild type value to 0.75–4.78 mg/ml, whereas *k*_*cat*_ decreased ~3–20,000 fold, and *k*_*cat*_/*K*_*m*_ ~8–187,500 fold. When compared to the wild type PG8fn, all mutants showed significant increases in △(△G) values (5.92–33.47 kJ/mol) (Table 2). In summary, the impact of mutations at position 94 caused drastic effects on the catalytic activity.

**Table 2:** Kinetic values of wild type PG8fn and its mutants ^a^.

Enzymes	*K*_*m*_ (mg/ml)	*k*_*cat*_ ^*b*^ (/s)	*k*_*cat*_/*K*_*m*_ (ml/mg/s)	△(△G)^c^ (kJ/mol)
Wild type	0.32 ± 0.01	60,000 ± 870	187,500	0
N94D	0.75 ± 0.01	15,000 ± 186	20,000	5.92
N94L	1.33 ± 0.04	6,200± 181	4,661	9.77
N94C	1.75 ± 0.05	3,100 ± 83	1,771	12.33
N94Q	2.38 ± 0.11	2,500 ± 111	1,050	13.33
N94G	2.83 ± 0.25	1,200 ± 108	424	16.11
N94S	3.78 ± 0.06	68 ± 1	18	24.47
N94A	4.74 ± 0.21	3 ± 0.1	0.6	33.47

^*a*^ The kinetic values are shown as means ± standard deviations (n = 3).

^*b*^ The *k*_*cat*_ values were calculated by considering the enzyme to be a monomeric form.

^*c*^ △(△G) = −RT·ln[(*k*_*cat*_/*K*_*m*_)_mut_/(*k*_*cat*_/*K*_*m*_)_wt_], where (*k*_*cat*_/*K*_*m*_)_mut_ and (*k*_*cat*_/*K*_*m*_)_wt_ are the *k*_*cat*_/*K*_*m*_ ratios of the mutant and wild type enzyme, respectively, R is the ideal gas constant, and T is the temperature in Kelvin.

The greatest change in *K*_*m*_ was observed for N94A, approximately a 14-fold increase. The *K*_*m*_ of N94A was higher than that of the mutants with bigger side chains, such as N94S and N94C, suggesting that longer side chains with non-optimal functioning in this positions still played a significant role in substrate binding. Accordingly, Asp and Leu side chains at this position changed the *K*_*m*_ but not as much as the smaller side chains; as a result, N94D and N94L had 1.3 and 3.2 fold increases in *K*_*m*_, respectively. The side chains of Asp and Asn are similar, but substitution of the negatively charged aspartic acid with asparagine might modify the charge of the local environment, and provide a repulsive environment for the highly negatively charged polygalacturonic acid. It may explain why N94D showed a much lower activity than the wild type. Since all mutants exhibited increased *K*_*m*_ values, it suggested that it is the acylamino group of Asn94 that plays almost an irreplaceable role in the binding of the substrate to the enzyme. Gln at 94 led to a conformational change in T3 loop. The side chain of Gln94 is too long and probably form hydrogen bonds in a disturbing way, therefore it does not stabilize the substrate to the correct place (6-fold increases in *K*_*m*_).

All the selected mutants also had an effect on *k*_*cat*_. Overall, the reduced catalytic efficiency was more due to the significant decreases in *k*_*cat*_ rather than in *K*_*m*_, although the *K*_*m*_ values were even dramatically increased, especially with N94A, which in practice completely abolished the endo-PG activity, leading to a ~187,500 fold decrease in catalytic efficiency. The mutants N94D and N94Q decreased the *k*_*cat*_ by 3 and 23 fold, and caused 8.3 and 177.5 fold decrease in catalytic efficiency, respectively, also indicating that the longer side chain of Gln is a key in the disturbing affect. The low increase in *K*_*m*_ by the mutation N94L indicated that Leu can function in binding, but the binding probably is incorrect since *k*_*cat*_ dropped dramatically.

According to the classical catalytic reaction scheme with a putative kinetically-controlled product released, the events in the mechanism can be defined as follows:

$$\text{E}+\text{S}\overset{\;k_{1}\;}{\underset{\;k_{-1}\;}{⇄}}\text{E}.\text{S}\overset{\;k_{2}\;}{\to }\text{E}.\text{P}\overset{\;k_{3}\;}{\to }\text{E}+\text{P}$$

The first presumptive conformation (B) would supposedly promote ligand binding to the active site, the second closed-conformation (C) would lock the bound ligand in the SBP and the third conformation (L) would allow product release. Although simple, this basic assumption is consistent with experimental evidence that has shown that T3 loop plays an important role in catalysis. A direct relation between T3 loop movement and catalytic properties involves the accurate positioning, and an intimate interaction between the substrate and the tip of T3 loop in +1 binding subsite. Different mutations in this position showed a range of effects on the catalytic parameters. By removing the hydrogen bonding contact between the tip of T3 loop and the substrate, the substitution of Asn94 by Ala led to an extremely reduced *k*_*cat*_ value. In the simulation of the N94A/pentagalacturonic acid complex, the pentagalacturonic acid was displaced from the docked position in the active site pocket suggesting the *k*_−1_ of the mutant N94A is much higher than that of the wild type enzyme. The specificity constant *k*_*cat*_ / *K*_*m*_ [*k*_*cat*_ / *K*_*m*_ = *k*_1_ *k*_2_ / (*k*_−1_ + *k*_2_)] is equal to *k*_1_ *k*_2_ / *k*_−1_ because *k*_−1_ is >> *k*_2_ in this situation. This illustrates that the side chain of Asn94 is not only involved in substrate binding but also stabilizes the bound substrate. It is supported by the fact that the mutant N94A has large *K*_*m*_ and low *k*_*cat*_. In a similar way Gly76 of pepsin, located in the middle of active site loop, was replaced by Ala, Val and Ser, and a lower flap catalytic efficiency was measured and interpreted as the result of lower flap flexibility [42].

Enzyme intramolecular mobility, conformational changes of loops in particular, plays a significant role in enzymatic catalysis [19–21, 40]. The loops may be required to close the substrate to active site. In general, the mutations changing the active site geometry and the amino acids interacting with the substrate may also often improve the catalytic efficiency [43], and the mutations may even have an effect on substrate binding due to a novel interaction via a water molecule [44]. Based on the present data, we conclude that T3 loop in GH28 endo-PG is a critical element of enzymatic functioning. The subsite is the narrowest part of the catalytic cleft, T3 and T1 loops lock the positions of the GalA moieties, and accurate binding in subsite −1/+1 determines how the catalytic residues can react with the substrate. Moreover, after the end of the reaction the opening of the loops permits release of the product and the beginning of a new catalytic cycle. However, our present knowledge does not provide any insight into the exact nature of this motion. T3 loop could assist product expulsion through an upward movement or by a lateral sliding motion like thumb-loop of GH11 xylanases [38] or flexible loop of ribonuclease A [45]. Further resolution of the crystal structure of PG8fn, experimentation and appropriate predictive modeling would be needed to achieve better understanding of this question.

## Conclusion

To probe the role of the mobile T3 loop of GH28 endo-PGs in substrate binding and catalysis, we simulated the structures of highly active PG8fn from *Achaetomium* sp. Xz8 and its complex with pentagalacturonic acid and verified the roles of the loop and the key residue Asn94. The results of MD simulation support the inverting mechanism of catalysis in PG8fn. Site-directed mutagenesis of Asn94 showed that this position is very sensitive to amino acid alterations. The disruption of hydrogen bonding interaction in the mutants displaced pentagalacturonic acid from the substrate binding pocket in the MD simulation. The failure to locate the substrate to the correct position close to the catalytic residues makes the catalytic reaction much slower. Basically, it is ascribed to the abolition of the original strong interaction between Asn94 and substrate in mutation complex structure, which in turn leads to a rather poor definition of the subsite +1. Overall, Asn94 on T3 loop is of great importance for substrate binding and plays an important role in stabilizing the substrate in such position that ensures the catalytic reaction happens efficiently.

## Supporting Information

S1 Fig Structural superimposition of seven molecules of CluPG1 from *Colletotrichum lupini* (PDB: 2IQ7). (DOCX)

S2 Fig Relative position of Asn91 in the structures of endoPG I from *S*. *purpureum*. The Apo-form structure (PDB: 1K5C), monogalacturonic acid-bound structure (PDB: 1KCC), and ternary complex structure containing two molecules of monogalacturonic acid (PDB: 1KCD) are indicated in green, yellow and purple, respectively.
(DOCX)

S3 Fig SDS-PAGE analysis of the purified recombinant PG8fn and its mutants. Lane M, the standard protein molecular weight markers; lanes 1, 3, 5, 7, 9, 11, 13, and 15, the purified wild type PG8fn and its mutants N94D, N94L, N94C, N94Q, N94G, N94S and N94A, respectively; lanes 2, 4, 6, 8, 10, 12, 14 and 16, the deglycosylated PG8fn and mutants N94D, N94L, N94C, N94Q, N94G, N94S and N94A, respectively.
(DOCX)

S1 Table Primers used in this study. (DOC)

S2 Table The predicted binding affinity of PG8fn and pentagalacturonic acid in all binding modes. (DOCX)
