# Supplementary material for: New Insights into the Role of T3 Loop in Determining Catalytic Efficiency of GH28 Endo-Polygalacturonases
Source: PLoS One. 2015 Sep 1;10(9):e0135413. doi: 10.1371/journal.pone.0135413 (PMC4556634; doi:10.1371/journal.pone.0135413)
Supplement: S1 Fig — (DOCX) [file pone.0135413.s001.docx]

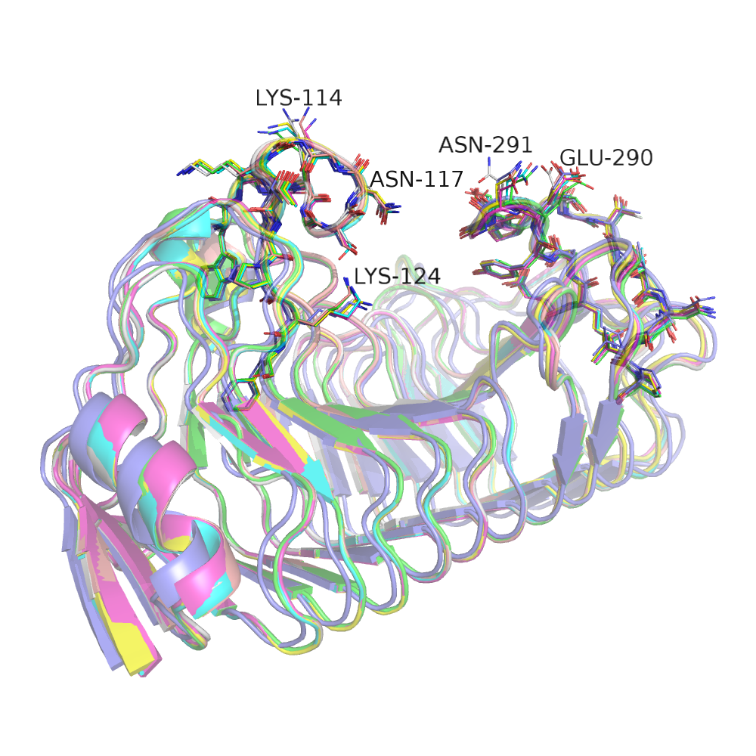


**S1 Fig.** Structural superimposition of seven molecules of CluPG1 from *Colletotrichum lupini* (PDB: 2IQ7).

T1 loop

T3 loop
