# Supplementary material for: New Insights into the Role of T3 Loop in Determining Catalytic Efficiency of GH28 Endo-Polygalacturonases
Source: PLoS One. 2015 Sep 1;10(9):e0135413. doi: 10.1371/journal.pone.0135413 (PMC4556634; doi:10.1371/journal.pone.0135413)
Supplement: S2 Fig — The Apo-form structure (PDB: 1K5C), monogalacturonic acid-bound structure (PDB: 1KCC), and ternary complex structure containing two molecules of monogalacturonic acid (PDB: 1KCD) are indicated in green, yellow and purple, respectively. (DOCX) [file pone.0135413.s002.docx]

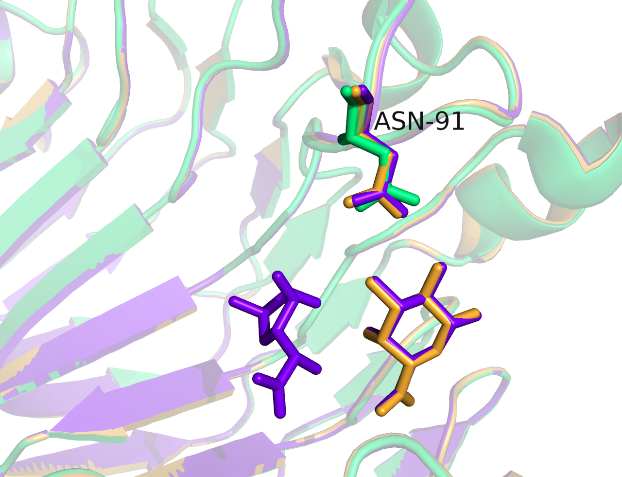


**S2 Fig.** Relative position of Asn91 in the structures of endoPG I from *S. purpureum*. The Apo-form structure (PDB: 1K5C), monogalacturonic acid-bound structure (PDB: 1KCC), and ternary complex structure containing two molecules of monogalacturonic acid (PDB: 1KCD) are indicated in green, yellow and purple, respectively.
