# Supplementary material for: New Insights into the Role of T3 Loop in Determining Catalytic Efficiency of GH28 Endo-Polygalacturonases
Source: PLoS One. 2015 Sep 1;10(9):e0135413. doi: 10.1371/journal.pone.0135413 (PMC4556634; doi:10.1371/journal.pone.0135413)
Supplement: S3 Fig — Lane M, the standard protein molecular weight markers; lanes 1, 3, 5, 7, 9, 11, 13, and 15, the purified wild type PG8fn and its mutants N94D, N94L, N94C, N94Q, N94G, N94S and N94A, respectively; lanes 2, 4, 6, 8, 10, 12, 14 and 16, the deglycosylated PG8fn and mutants N94D, N94L, N94C, N94Q, N94G, N94S and N94A, respectively. (DOCX) [file pone.0135413.s003.docx]

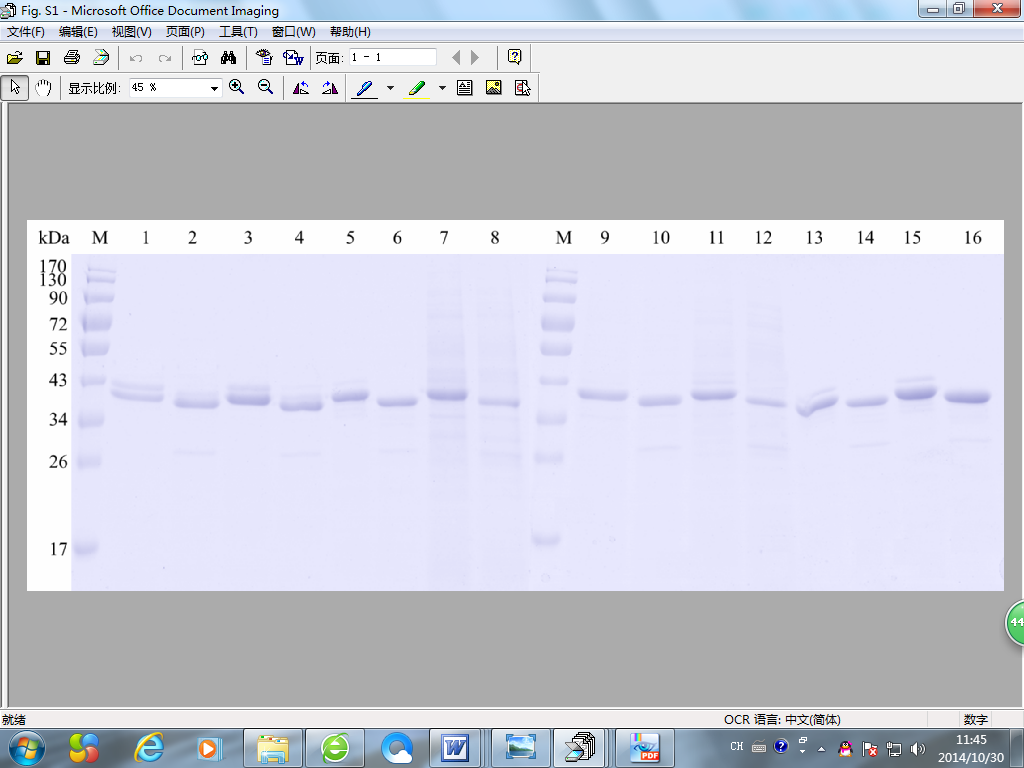


**S3 Fig.** SDS-PAGE analysis of the purified recombinant PG8fn and its mutants. Lane M, the standard protein molecular weight markers; lanes 1, 3, 5, 7, 9, 11, 13, and 15, the purified wild type PG8fn and its mutants N94D, N94L, N94C, N94Q, N94G, N94S and N94A, respectively; lanes 2, 4, 6, 8, 10, 12, 14 and 16, the deglycosylated PG8fn and mutants N94D, N94L, N94C, N94Q, N94G, N94S and N94A, respectively.
