# Supplementary material for: New Insights into the Role of T3 Loop in Determining Catalytic Efficiency of GH28 Endo-Polygalacturonases
Source: PLoS One. 2015 Sep 1;10(9):e0135413. doi: 10.1371/journal.pone.0135413 (PMC4556634; doi:10.1371/journal.pone.0135413)
Supplement: S1 Table — (DOC) [file pone.0135413.s004.doc]

**S1 Table** Primers used in this study

| Enzyme | Oligonucleotide sequence (5′→3′)*^a^* |
| --- | --- |
| N94A-F | ACCAAGGGTAGCGCTGGCGGCAAGAAGAAG |
| N94A-R | CTTCTTGCCGCCAGCGCTACCCTTGGTGT |
| N94C-F | ACCAAGGGTAGCTGTGGCGGCAAGAAGAAGC |
| N94C-R | CTTCTTGCCGCCACAGCTACCCTTGGTGTC |
| N94G-F | ACCAAGGGTAGCGGTGGCGGCAAGAAGAAG |
| N94G-R | CTTCTTGCCGCCACCGCTACCCTTGGTGT |
| N94S-F | ACCAAGGGTAGCTCCGGCGGCAAGAAGAAG |
| N94S-R | CTTCTTGCCGCCGGAGCTACCCTTGGTGT |
| N94L-F | ACCAAGGGTAGCTTGGGCGGCAAGAAGAAGC |
| N94L-R | CTTCTTGCCGCCCAAGCTACCCTTGGTGTC |
| N94Q-F | ACCAAGGGTAGCCAAGGCGGCAAGAAGAAGC |
| N94Q-R | CTTCTTCTTGCCGCCTTGGCTACCCTTGGTGT |
| N94D-F | ACCAAGGGTAGCGACGGCGGCAAGAAGAAG |
| N94D-R | CTTCTTGCCGCCGTCGCTACCCTTGGTGT |

*^a^*The mutation sites are underlined.

T3 loop

T1 loop
