# Supplementary material for: New Insights into the Role of T3 Loop in Determining Catalytic Efficiency of GH28 Endo-Polygalacturonases
Source: PLoS One. 2015 Sep 1;10(9):e0135413. doi: 10.1371/journal.pone.0135413 (PMC4556634; doi:10.1371/journal.pone.0135413)
Supplement: S2 Table — (DOCX) [file pone.0135413.s005.docx]

**S2 Table** The predicted binding affinity of PG8fn and pentagalacturonic acid in all binding modes

| Modes | Affinity  (kcal/mol) | Distance from best mode (RMSD) | |
| --- | --- | --- | --- |
|  |  | Lower | Upper |
| 1 | −9.3 | 0.000 | 0.000 |
| 2 | −9.3 | 2.489 | 4.362 |
| 3 | −9.3 | 1.863 | 3.280 |
| 4 | −9.2 | 4.595 | 10.356 |
| 5 | −9.0 | 1.972 | 4.570 |
| 6 | −8.9 | 1.946 | 4.460 |
| 7 | −8.9 | 2.157 | 4.308 |
| 8 | −8.9 | 2.194 | 13.688 |
| 9 | −8.8 | 1.694 | 2.689 |
| 10 | −8.8 | 5.972 | 12.821 |
| 11 | −8.8 | 5.980 | 13.156 |
| 12 | −8.7 | 1.901 | 4.515 |
| 13 | −8.7 | 5.263 | 17.968 |
| 14 | −8.7 | 1.822 | 14.539 |
| 15 | −8.7 | 1.821 | 14.859 |
| 16 | −8.6 | 1.674 | 14.039 |
| 17 | −8.6 | 1.852 | 14.072 |
| 18 | −8.4 | 2.197 | 13.435 |
| 19 | −8.3 | 5.930 | 12.981 |
| 20 | −8.0 | 6.318 | 16.482 |
